# Personalized upper limb training combined with anodal-tDCS for sensorimotor recovery in spastic hemiparesis: study protocol for a randomized controlled trial

**DOI:** 10.1186/s13063-017-2377-6

**Published:** 2018-01-04

**Authors:** Mindy F. Levin, Melanie C. Baniña, Silvi Frenkel-Toledo, Sigal Berman, Nachum Soroker, John M. Solomon, Dario G. Liebermann

**Affiliations:** 1 School of Physical and Occupational Therapy, Faculty of Medicine, McGill University, Montreal, QC Canada; 2 Center for Interdisciplinary Research in Rehabilitation (CRIR), Montreal, QC Canada; 3 Department of Physical Therapy, Stanley Steyer School of Health Professions, Sackler Faculty of Medicine, Tel Aviv University, Tel Aviv, Israel; 4 Department of Neurological Rehabilitation, Loewenstein Hospital, Ra’anana, Israel; 5 Department of Industrial Engineering and Management, Ben-Gurion University of the Negev, Beer-Sheva, Israel; 6 Sackler Faculty of Medicine, Tel Aviv University, Tel Aviv, Israel; 7 Department of Physiotherapy, School of Allied Health Sciences (SOAHS), Manipal University, Manipal, Karnataka India; 8 School of Physical and Occupational Therapy, McGill University, 3654 Promenade Sir William Osler, Montreal, QC H3S 1Y5 Canada

**Keywords:** Stroke, Spasticity, Spatial threshold, tDCS, Neurorehabilitation

## Abstract

**Background:**

Recovery of voluntary movement is a main rehabilitation goal. Efforts to identify effective upper limb (UL) interventions after stroke have been unsatisfactory. This study includes personalized impairment-based UL reaching training in virtual reality (VR) combined with non-invasive brain stimulation to enhance motor learning. The approach is guided by limiting reaching training to the angular zone in which active control is preserved (“active control zone”) after identification of a “spasticity zone”. Anodal transcranial direct current stimulation (a-tDCS) is used to facilitate activation of the affected hemisphere and enhance inter-hemispheric balance. The purpose of the study is to investigate the effectiveness of personalized reaching training, with and without a-tDCS, to increase the range of active elbow control and improve UL function.

**Methods:**

This single-blind randomized controlled trial will take place at four academic rehabilitation centers in Canada, India and Israel. The intervention involves 10 days of personalized VR reaching training with both groups receiving the same intensity of treatment. Participants with sub-acute stroke aged 25 to 80 years with elbow spasticity will be randomized to one of three groups: personalized training (reaching within individually determined active control zones) with a-tDCS (group 1) or sham-tDCS (group 2), or non-personalized training (reaching regardless of active control zones) with a-tDCS (group 3). A baseline assessment will be performed at randomization and two follow-up assessments will occur at the end of the intervention and at 1 month post intervention. Main outcomes are elbow-flexor spatial threshold and ratio of spasticity zone to full elbow-extension range. Secondary outcomes include the Modified Ashworth Scale, Fugl-Meyer Assessment, Streamlined Wolf Motor Function Test and UL kinematics during a standardized reach-to-grasp task.

**Discussion:**

This study will provide evidence on the effectiveness of personalized treatment on spasticity and UL motor ability and feasibility of using low-cost interventions in low-to-middle-income countries.

**Trial registration:**

ClinicalTrials.gov, ID: NCT02725853. Initially registered on 12 January 2016.

**Electronic supplementary material:**

The online version of this article (doi:10.1186/s13063-017-2377-6) contains supplementary material, which is available to authorized users.

## Background

Stroke is a leading cause of long-term disability. Up to 85% of patients with sub-acute stroke present chronic upper limb (UL) sensorimotor deficits [1]. While post-stroke UL recovery has been a major focus of attention, efforts to identify effective rehabilitation interventions have been unsatisfactory. This study focuses on the delivery of personalized impairment-based UL training combined with low-cost state-of-the-art technology (non-invasive brain stimulation and commercially available virtual reality, VR) to enhance motor learning, which is becoming more readily available worldwide.

A major impairment following stroke is spasticity, leading to difficulty in daily activities and reduced quality of life [2]. Studies have identified that spasticity relates to disordered motor control due to deficits in the ability of the central nervous system to regulate motoneuronal thresholds through segmental and descending systems [3, 4]. In the healthy nervous system, the motoneuronal threshold is expressed as the “spatial threshold” (ST) or the specific muscle length/joint angle at which the stretch reflex and other proprioceptive reflexes begin to act [5–7]. The range of ST regulation in the intact system is defined by the task-specific ability to activate muscles anywhere within the biomechanical joint range of motion (ROM). However, to relax the muscle completely, ST has to be shifted outside of the biomechanical range [8].

**Fig. 1:**
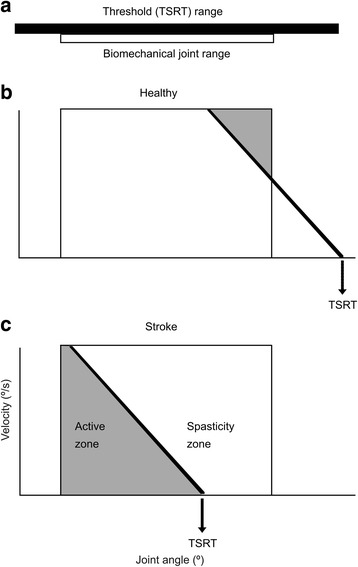
Spatial thresholds (STs) in healthy and stroke participants. **a** The tonic stretch reflex threshold (TSRT) can be regulated throughout a range (filled bar) that exceeds the biomechanical range of the joint (open bar). Relaxation and active force can be produced at any angle within the biomechanical range. **b** The intersection of the diagonal line with the zero-velocity line defines the TSRT. In healthy subjects, TSRT lies outside of the biomechanical range of the joint (arrow) during the relaxed state. **c** In patients with stroke, TSRT may lie within the biomechanical range in the relaxed state, defining the joint angle at which spasticity begins to appear (spasticity zone). In the other joint ranges, spasticity is not present (active zone)

After stroke, the ability to regulate STs is impaired [3] such that the upper angular limit of ST regulation occurs within the biomechanical range of the joint resulting in spasticity (spasticity zone). Thus, resistance to stretch of the relaxed muscle has a spatial aspect in that it occurs within the defined spasticity zone. In other joint ranges, spasticity is not present and normal reciprocal muscle activation can occur (active control zone; [4] Fig. 1). This theory-based intervention investigates whether recovery of voluntary movement is linked to recovery of ST control.

We also consider that inter-hemispheric balance is disrupted after stroke, interfering with recovery. UL motor function depends on the modulation of inter-hemispheric inhibition between cortical areas via transcallosal projections [9, 10] and descending projections to fingers, hand and arm [11]. Unilateral hemispheric damage reduces activity in the affected hemisphere while activity in the unaffected hemisphere increases [12], becoming more dominant. UL recovery may relate to rebalancing of inter-hemispheric inhibition [13] using, for example, anodal transcranial direct current stimulation (a-tDCS) over the affected hemisphere [14, 15]. a-tDCS is considered a safe technique with transient adverse effects, such as slight scalp itching or tingling and/or mild headaches, that are not expected to impede the patient’s ability to participate in the training protocol [16].

The underlying idea of this proposal is that recovery of voluntary movement is tightly linked to the recovery of threshold control. We propose an intervention that combines current knowledge about motor learning and disorders in ST control. The intervention involves personalized UL reach training designed according to the spatial structure of motor deficits of an individual, with excitatory a-tDCS over the sensorimotor areas of the affected hemisphere.

## Methods

### Aim, design and setting

**Fig. 2:**
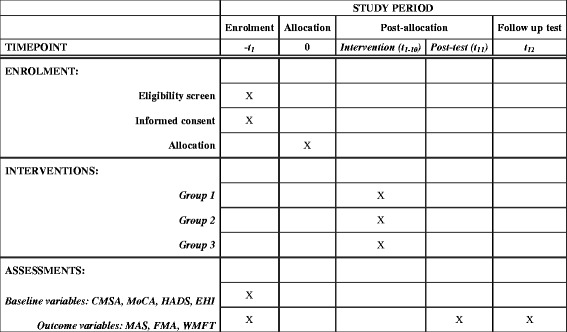
Schedule of enrollment, interventions and assessments. Standard Protocol Items: Recommendations for Interventional Trials (SPIRIT) flow diagram of study enrollment, interventions and assessments

We hypothesize that in patients with sub-acute spastic hemiparesis after stroke, practice restricted to the active control zone (personalized training) combined with excitatory a-tDCS will improve control over the range of ST regulation in the elbow and improve UL kinematic and clinical performance measures compared to non-personalized training with a-tDCS and personalized training with sham-tDCS. We propose a single-blind, parallel, three-group, randomized controlled trial (RCT; allocation ratio 1:1:1) involving personalized therapeutic intervention to improve control over the range of regulation of STs with the application of excitatory a-tDCS, which, under generally facilitatory conditions (e.g., motivating patients with VR games), may strengthen remaining connections and help restore inter-hemispheric balance. The Standard Protocol Items: Recommendations for Interventional Trials (SPIRIT) flow diagram of study enrollment, interventions and assessments is shown in Fig. 2. In addition, the implementation of such training programs in a low-to-middle-income country will be investigated by including four academic rehabilitation centers in Canada, Israel and India.

### Participants and recruitment

Sixty people with sub-acute spastic hemiparesis after stroke will be recruited during their hospitalization in rehabilitation centers. In order to achieve adequate participant enrollment, constant chart review of newly admitted patients will be done. Individuals will be included if they have/are: (1) a stroke in the middle cerebral territory, confirmed by magnetic resonance imaging/computed tomography (MRI/CT), and medically stable; (2) aged 25–80 years; (3) in the sub-acute stage of stroke (3 weeks to 6 months post stroke); (4) arm paresis (2–6/7 on the Chedoke-McMaster Stroke Assessment arm score, CMSA [17]) and able to perform at least 30° elbow flexion and extension; (5) elbow flexor and/or extensor spasticity (>1 + out of 4 on the Modified Ashworth Scale, MAS [18, 19]); and (6) able to provide informed consent. Exclusion criteria are: (1) other major neurological or neuromuscular/orthopedic problems or pain interfering with results’ interpretation; (2) medical co-morbidities such as HIV/AIDS, etc.; (3) major cognitive deficits (˂ 20 on the Montreal Cognitive Assessment, MoCA [20]); (4) history of psychiatric disorders, alcohol or drug abuse, seizures, migraines, metal in the cranium, and cochlear and cardiac implants; (5) taking medications (e.g., anti-epileptic and psychoactive drugs) that could affect brain activity [21, 22]. Criteria for discontinuation include participant request or adverse events.

Eligibility will be established by a blinded research coordinator. Participants will provide informed consent and be allocated to one of three treatment groups (*n* = 20 per group) by permuted block randomization, balanced for age (25–50 years/51–80 years) and chronicity (3–12 weeks/13–24 weeks). Participants will receive a training and evaluation schedule, and participation will be monitored by the study coordinator, who will also provide reminders to attend scheduled activities. Travel expenses will be remunerated for follow-up visits.

The sample size is based on preliminary data showing that the mean change in elbow-flexor ST angle was 10° in stroke subjects with spasticity who underwent 2 weeks of transcutaneous electrical nerve stimulation treatment compared to 2° after sham stimulation (unpublished data). Considering an *α* level of 5% and a 95% power (effect size = 2.23) to detect differences using a mixed-design 3 × 3 analysis of variance (ANOVA) (G*Power 3.1.1), the minimal sample size is 13 subjects per group. Sample size was increased to 18 per group to have an equal number of subjects per group per site (six per group, per site). We will recruit 20 subjects per country overall considering a dropout rate of 10–15% for a final cohort of 60 subjects.

Prior to the start of recruitment of participants, standardized procedures will be established to ensure consistency across sites. Intervention therapists will receive training guidelines and evaluators will receive screening and clinical assessment documents. In addition, all team members directly involved in data collection will have face-to-face training on data collection protocols. Quarterly online meetings will be scheduled to maintain adherence to the protocols across sites.

### Intervention

Participants will enroll in a 10-day training program over two consecutive weeks (5 days per week). Outcome evaluators, statistical analysts and care providers will be blind to group assignment but not the intervention therapists, given the nature of the intervention. Intervention therapists will receive allocation information from the study coordinator via coded email. Participants will be randomly allocated as follows: (group 1) personalized training (reaching within determined active control zones) and a-tDCS over the affected hemisphere; (group 2) non-personalized training (reaching in both spasticity and active control zones; full active ROM) and a-tDCS over the affected hemisphere; and (group 3) personalized training and sham-tDCS.

Prior to the reaching training, elbow-flexor STs in the hemiparetic arm will be determined in each participant to identify the active control zone, using the Montreal Stretch Reflex Threshold device (MSRT; [23] see below). For the personalized training groups (groups 1 and 3), elbow extension will be restricted to the active control range using a range-restriction elbow brace (Breg Inc., Carlsbad, CA, USA) blocking elbow extension beyond the permitted ST. Participants in the non-personalized training group will wear the brace, but elbow ROM will not be restricted. All participants will have partial arm-weight support with a sling during the training to avoid fatigue. Since a goal of the training is to increase isolated elbow extension, the sling will be placed so that the weight of the upper arm and shoulder is supported without restricting elbow movements. The support will be provided throughout the training sessions for all subjects so that training parameters are equivalent across groups and sites.

**Fig. 3:**
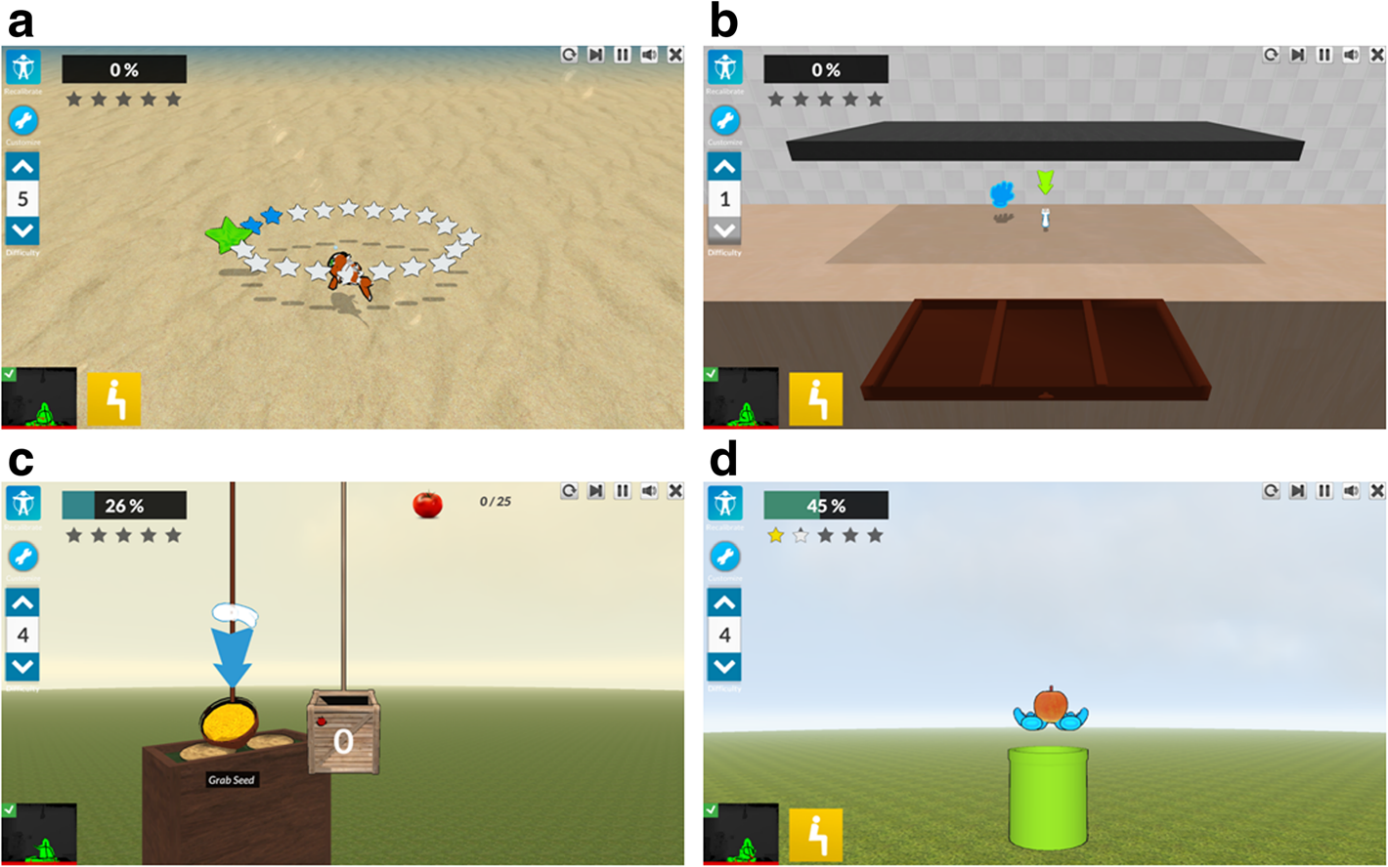
Jintronix virtual reality (VR) games used in the intervention. **a** Fish Frenzy game requires the player to trace a three-dimensional (3D) trajectory by moving a fish on the screen in different shapes. **b** Kitchen Cleanup game requires forward reaching towards kitchen cutlery and returning them to shelves and drawers. **c** Garden Grab game requires lateral reaching while planting seeds, harvesting and transferring tomatoes to baskets. **d** Catch, Carry, Drop game requires bilateral coordination while catching apples, carrying and dropping them into a container

During reaching training, participants will sit on an armless chair and make discrete arm reaches towards targets displayed at various locations in the three-dimensional (3D) workspace with unrestrained trunk movement. Training will be guided by a physical or occupational therapist for 50 min (actual movement time) per day. Four interactive games from the Jintronix VR system (Montreal, QC, Canada) will be viewed on a large screen. Three activities will involve unimanual movement and one will involve a bimanual activity (Fig. 3). The total amount of movement time each training day will be recorded by the Jintronix system. Treatments will be matched for both duration and intensity across sites.

#### Calibration and game progression

In the personalized practice group, the Jintronix system will be calibrated prior to each training session according to the individual’s ability to reach within the active zone only, whereas in the non-personalized practice group the calibration will be done based on the individual’s full active ROM. In this way, the calibration procedure is used to set and progress the sagittal reaching distance in each game from near to far according to the length of the subject’s effective sagittal reaching distance. Success in each reaching task depends on a combination of reaching distance, speed and precision according to Fitts’ law [24]. Thus, difficulty levels of each activity will be increased by altering the location of the virtual targets and by decreasing the allowable movement time to complete each reach (i.e., by increasing movement speed), while target size remains the same. Levels will be progressed when the participant reaches at least 95% success rate in three rounds. Game difficulty will be progressed according to the Challenge Point Theory of motor learning [25]. This theory suggests that learning is enhanced by optimally challenging the individual through manipulation of task difficulty according to their motor skill level and cognitive capacity. Since learning is also affected by performance-related feedback, participants will receive concomitant visual and auditory Knowledge of Results about task success when movements are made accurately and within a specified lapse of time programmed by the therapist. Participants will also receive negative Knowledge of Performance about the use of compensatory trunk movements during reaching through the Jintronix system. Thus, if the participant leans their trunk more than 5° in the sagittal direction, a yellow square will appear on the screen and a reach success will not be registered. The 5° trunk-flexion range was chosen since it corresponds to the cutoff value identified in Subramanian et al. [26] as indicating compensatory trunk movement. Regardless of the training zone and difficulty level, all participants in each group will follow the same training guidelines and complete the 50 min of active movement time.

A-tDCS (Soterix, New York, NY, USA) will be provided by a constant direct current stimulator at an intensity of 1.5 mA, applied via saline-soaked surface electrodes (5 cm × 7 cm) on the scalp. Stimulation will occur during the initial 30 min of the 50-min training session. The anode will be placed over the sensorimotor areas of the affected hemisphere (placed over C3/C4 using the electroencephalogram (EEG) 10–20 referencing system), while the cathode will be placed on the contralateral supraorbital area. For the sham stimulation, the same electrode placement will be followed, but the device pre-programmed sham setting will apply stimulation only for the first minute. In the first 30 s, a stimulation ramp-up is expected to cause a mild itching sensation underneath the anodal electrode. This sensation is expected to fade between 40 and 60 s, at the end of which the intensity reaches zero level. No stimulation will be provided for the rest of the practice session.

As this trial intervention is carried out in different centers, the adherence to the intervention will be ensured by recording the frequency and the intensity of the games on standardized documents, supervision by the lead investigator at each site and supervision by the co-principal investigators (PIs) via regular meetings.

### Study outcomes

Outcome measures will be documented before (Pre), after 10 days of intervention (Post) and 1 month after intervention end (Follow-up). Measures will be used to obtain an overall clinical profile of arm and hand impairment and function. Arm motor recovery will be evaluated at two levels of the International Classification of Function: Body Structure and Function (motor impairment) and Activity [27]. The primary outcome will be the ST angle and the ratio between the range of the biceps spasticity zone and the full biomechanical elbow-extension ROM.

#### Assessment of spasticity and active control zones

**Fig. 4:**
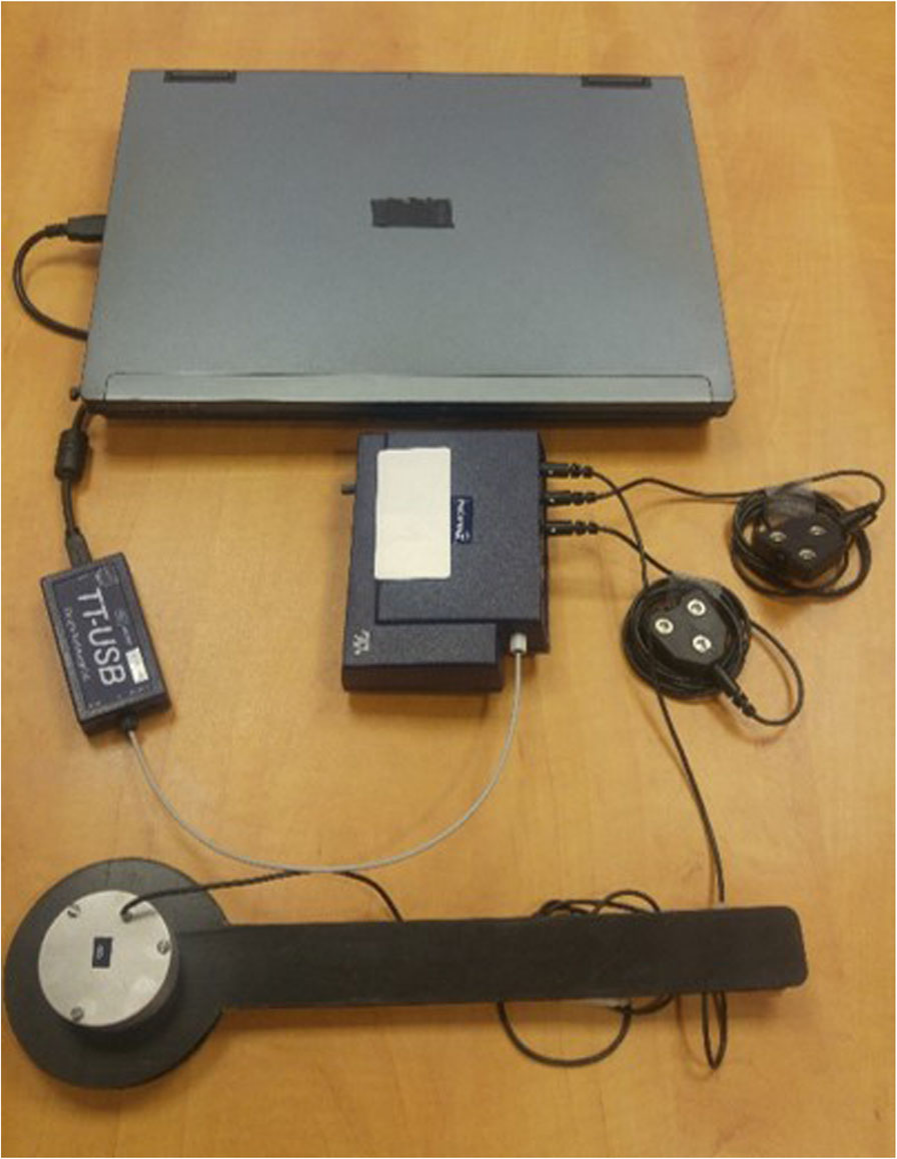
Montreal Stretch Reflex Threshold (MSRT) device. The MSRT is a portable, clinical device consisting of a two-channel electromyography (EMG) system, an electrogoniometer and dedicated software implemented on a laptop computer

Since active elbow extension (active control zone) is limited by the location of the ST in the angular range of the elbow, the ST angle of the elbow flexors (biceps brachii, BB) will be measured. This will allow us to determine the range of angles in which BB cannot be relaxed (spasticity zone) and the range in which reciprocal agonist/antagonist muscle activation is possible (active control zone). The ST angle will be defined using the MSRT, a portable clinical device consisting of a two-channel electromyography (EMG) system (Procomp 5, Thought Technology, Montreal, QC, Canada), an electrogoniometer (servo-type rotational-position potentiometer P2200; Novotechnik U.S. Inc., Southborough, MA, USA) and dedicated software implemented on a laptop computer (Fig. 4). The MSRT has moderate-to-good intra- and inter-evaluator reliability for the measurement of elbow spasticity in post-stroke participants [23].

BB and triceps brachii (TB) EMG recordings will be obtained with surface electrodes (Ambu® Blue Sensor P, Ballerup, Denmark), from motor points indicated in the Surface ElectroMyoGraphy for the Non-Invasive Assessment of Muscles (SENIAM) guidelines [28]. EMG signals will be band-pass filtered (10–1000 Hz), amplified (×500) and sampled at 2048 samples/s. Seated participants will perform a maximal voluntary contraction of the elbow flexors to adjust EMG gain and initial elbow angle will be specified. Then, 20 stretches will be performed manually by stretching the elbow from flexion (approximately 50^o^) to full extension. Participants will be instructed to relax completely without assisting or resisting the angular displacement. Adequate rest (10 s) will be allowed between stretches to minimize effects of fatigue and muscle thixotropy [29]. Before each stretch, a randomly assigned speed, equally distributed between slow, moderate and fast velocities, will be indicated by an auditory signal from the MSRT to avoid the participant anticipating the upcoming stretch. Each evaluation will last for approximately 20 min. At the end of each stretch, MSRT identifies the angle at which the EMG begins, called the dynamic stretch reflex threshold (DSRT) [23]. At the completion of at least 20 correct stretches, a linear regression line is computed through the DSRTs on the angular velocity/displacement plot from which the correlation coefficient (*r*^2^), slope and x-axis intercept are determined. The x-axis intercept corresponds to the tonic stretch reflex threshold (TSRT) angle. A low TSRT angle corresponds to a high level of spasticity.

#### Arm motor impairment

Voluntary arm motor ability and coordination will be evaluated using the valid and reliable Fugl-Meyer Assessment (FMA) [30–32] that evaluates reflexes, volitional movements and rapid alternating movements. UL scores range from 0 to 66. Sensation (light touch), kinesthesia and passive ROM will be evaluated using FMA scales.

Clinical spasticity in elbow flexors and extensors will be measured with the MAS [18, 19] on six-point ordinal scales (0–1, 1 +, 2–4). The MAS has poor-to-good inter-rater reliability [19, 33, 34].

#### Arm motor function

Arm functional ability will be assessed with the six-item Streamlined Wolf Motor Function Test (S-WMFT) consisting of (1) speed of completion and (2) movement quality scores. Items 1 and 2 are activities involving elbow and shoulder movements and items 3 to 6 are functional tasks. Each item has a maximal time of 120 s and the mean will be reported. In the case of inability to complete a task, a time score of 121 s will be assigned [35]. Functional ability is rated on a six-point scale from 0 to 5 where 0 indicates no attempt to move the affected UL and 5 indicates normal movement. Mean scores will be reported. The S-WMFT has good concurrent (Spearman’s *ρ* = 0.69) and predictive validity (Spearman’s *ρ* = 0.68) with FMA. Responsiveness, as assessed by the standard response mean, was 0.41 [36].

#### Kinematic recording and analysis of reach-to-grasp task

A kinematic assessment of a common daily reach-to-grasp task requiring different amounts of elbow extension will be recorded with a room- and body-calibrated five-sensor wireless electromagnetic tracking system G4 (Polhemus, Colchester, VT, USA; RMS static accuracy 0.20 cm for position and 0.5° for orientation at a distance of 1 m from the transmitter). Each sensor is tracked at a rate of 120 Hz providing six degrees of freedom (three Cartesian and three polar). Sensors will be placed on the index finger metacarpo-phalangeal joint, the proximal third of the dorsal forearm, the mid-lateral surface of the arm, the mid-point of the superior-lateral border of the acromion, and mid-sternum.

Participants will sit in front of a table on a standard chair with no armrests. Arm position will be adjusted so that in the initial position the elbow will be in 30° flexion alongside the body and feet will be supported on the floor. Participants will be instructed to reach to grasp a 6-cm diameter cone “as fast and as precisely as possible,” hold or touch the cone for 2 s, lift the cone or arm towards the chin and then return it to the table. A mid-sagittal reference frame will define four target locations. Targets 1 and 2 will be placed at two thirds and full arm’s length in the mid-sagittal plane, respectively, for which different levels of elbow extension are required. Targets 3 and 4 will be placed at approximately 20 cm to the right and left of Target 2. Arm length will be measured from the medial axillary border to the distal wrist crease with the elbow extended [37]. After several initial practice trials (two trials/target), participants will perform two sets of 40 trials (2 × 10 trials × 4 targets = 80 movements; randomized), in approximately 1 h. Rest between sets and trials will be provided.

Motor behavior will be measured at two levels: endpoint performance and arm movement quality [38]. Endpoint performance includes peak speed; trajectory straightness (index of curvature, IC) [39] where IC > 1.57 describes a semi-circle; and smoothness calculated as the number of velocity peaks in the tangential velocity profile.

For movement quality, joint angles involved in reaching will be measured including shoulder flexion, abduction/adduction and rotation, elbow extension, wrist supination and extension using Euler angles based on standard 3D kinematic reconstruction [40]. Analysis of inter-joint coordination, compensations and pathological synergies will include consideration of trunk and arm plane motion [41].

#### Lesion type, size and location

In a secondary analysis, the relationship between specific lesion type, size and location and treatment outcomes will be assessed.

### Data management

The Oversight Committee will consist of the PIs and one other individual not associated with the trial. The Data Monitoring and Management Committee will be led by an individual who will ensure that all sites upload data from each phase of the trial to a secured, online data repository. Clinical, kinematic and EMG data will be coded for confidentiality. Adverse events due to a-tDCS, training or testing paradigms will be reported to the Ethics Committee at each site by filling out an incident report form. The Ethics Committee will then meet to discuss possible solutions.

### Statistical analyses

Descriptive statistics will be used to highlight main demographic and clinical characteristics of participants. After verification for linearity, normality and homoscedasticity, parametric mixed-model ANOVAs will be used to determine which stimulation and practice modality combinations lead to greater improvements. The mixed-model analysis will include one between-subject factor – group with three levels (personalized training + a-tDCS, non-personalized training + a-tDCS and personalized training + sham-tDCS), and one within-subject factor – time with three levels (Pre, Post and Follow-up). Multiple linear regression analyses on pooled data will identify relationships between primary outcomes and changes in clinical and kinematic measures. If assumptions for parametric tests are not met, non-parametric tests will be substituted. Results will be analyzed based on intention-to-treat principles. Individual participation in other in-patient services will be documented (physiotherapy, occupational therapy, speech therapy, social work, recreation, other) and compared between sites to account for effects of usual-care exposure. Results will be disseminated to individuals, user groups, health care and research professionals at trial completion.

### SPIRIT Checklist

Please see Additional file 1 for the SPIRIT Checklist [42].

## Discussion

Based on the identification of disorders in stretch reflex threshold control in each subject, we will implement a personalized VR-enhanced UL training program with and without a-tDCS. Our innovative approach aims to maximize the recovery potential of each individual by taking into consideration motor impairment as defined by Threshold Control Theory [8] and training based on established principles of motor learning that are assumed to maximize neural plasticity. Optimization of UL motor function is expected due to timing the intervention during the sub-acute phase of stroke when the most rapid recovery occurs [43].

During the design phase of the RCT, low-cost equipment was specifically chosen to enable accessibility for institutions in any country regardless of income level. To date, all centres successfully built the study set-up and simultaneously started implementation of the study.

## Trial status

The RCT recruitment is planned from June 2016 to December 2018. Data analysis and evaluation will be performed subsequently. Final results of this study will be published.

## Additional file

Additional file 1: SPIRIT 2013 Checklist: recommended items to address in a clinical trial protocol and related documents. (PDF 152 kb)

## Abbreviations

a-tDCS: Anodal transcranial direct current stimulation
BB: Biceps brachii
CMSA: Chedoke-McMaster Stroke Assessment
CT: Computed tomography
DSRT: Dynamic stretch reflex threshold
EEG: Electroencephalogram
EMG: Electromyography
FMA: Fugl-Meyer Assessment
BUT: Modified Ashworth Scale
Mocha: Montreal Cognitive Assessment
MRI: Magnetic resonance imaging
MSRT: Montreal Stretch Reflex Threshold device
ROM: Range of motion
Senior: Surface ElectroMyoGraphy for the Non-Invasive Assessment of Muscles
ST: Spatial threshold
S-WMFT: Streamlined Wolf Motor Function Test
TB: Triceps brachii
TSRT: Tonic stretch reflex threshold
UL: Upper limb
VR: Virtual reality
